# TNF-Like Ligand 1 Aberrance Aggravates Nonalcoholic Steatohepatitis via M1 Macrophage Polarization

**DOI:** 10.1155/2021/3877617

**Published:** 2021-12-31

**Authors:** Yuxin Luo, Jinbo Guo, Wenxiu Jia, Mengyao Wu, Fengrong Yin, Guochao Niu, David Q. Shih, Stephan R. Targan, Xiaolan Zhang

**Affiliations:** ^1^Department of Gastroenterology, The Second Hospital of Hebei Medical University, Hebei Medical University, Shijiazhuang, China; ^2^F. Widjaja Foundation Inflammatory Bowel and Immunobiology Research Institute, Cedars-Sinai Medical Center, Los Angeles, USA

## Abstract

Nonalcoholic steatohepatitis (NASH) is a progressive, chronic liver disease worldwide which imposes a large economic burden on society. M1/M2 macrophage balance destruction and recruitment of mononuclear immune cells to the liver play critical roles in NASH. Several studies have shown that the expression of TNF-like ligand 1 aberrance (TL1A) increased in macrophages associated with many inflammatory diseases, for example, inflammatory bowel disease, primary biliary cholangitis, and liver fibrosis. One recent research showed that weight, abdominal adipose, and liver leptin, one of the critical fat cytokines, were reduced in *TL1A* knockout mice. However, the functional and molecular regulatory mechanisms of TL1A on macrophage polarization and recruitment in NASH have yet to be clarified. The authors found that high fructose high fat diet and methionine-choline deficiency diet induced the expression of TL1A in macrophages of liver tissue from murine NASH models. Myeloid-specific TL1A overexpressed mice showed exacerbated steatohepatitis with increased hepatic lipid accumulation, inflammation, liver injury, and apoptosis. M1 macrophages' infiltration and the production of proinflammatory and chemotactic cytokines increased in liver of NASH mouse models with myeloid-specific TL1A overexpressed. Furthermore, this paper revealed that bone marrow-derived macrophages and Kupffer cells with overexpression of TL1A exacerbated the lipid accumulation and expression of proinflammatory factors in the murine primary hepatocytes after free fatty acid treatment *in vitro*. In conclusion, TL1A-mediated M1-type macrophage polarization and recruitment into the liver promoted steatohepatitis in murine NASH.

## 1. Introduction

Nonalcoholic steatohepatitis (NASH) is an aggressive form of nonalcoholic fatty liver disease (NAFLD), which characterized by excessive fatty accumulation in liver, cell inflammation, and liver fibrosis. Ultimately, cirrhosis increases hepatocellular carcinoma (HCC) development risks [1, 2]. With the improvement of living standards and change of dietary habits, the morbidity of NASH gradually increases. Patients are younger. It has become a major healthcare burden worldwide [3, 4]. However, the pathogenesis of NASH is multifactorial and remains incompletely defined. Therefore, the investigation of NASH is of great necessity and significance.

According to “multiple-hit” hypothesis, which is thought to be the classical theory of NAFLD, oxidative stress and inflammation are the two pivotal mechanisms [5]. As the key player of the innate immune system, macrophages play an important role in NASH by regulating inflammatory responses. Hepatocytes with redundant lipid accumulation could induce peroxidation to activate macrophages and promote M1 macrophage polarization. Macrophage polarization to M1 phenotype is associated with the production of proinflammatory cytokines, for example, tumor necrosis factor- (TNF-) *α*, interleukin- (IL-) 1*β*, and IL-6. Meanwhile, these proinflammatory cytokines could aggravate steatosis and promote apoptosis by inhibiting the outflow of cholesterol in hepatocytes. Proinflammatory macrophages could also recruit mononuclear macrophages expressed C-C chemokine receptor 2 (CCR2) and C-X-C chemokine receptor 2 (CXCR2) in the blood into the liver and accelerate inflammatory response by secreting C-C chemokine ligand 2 (CCL2) and chemokine C-X-C ligand 1 (CXCL1).

Several studies showed that TNF-like ligand 1 (TL1A), a member of the tumor necrosis factor superfamily (TNFSF) of ligands, was implicated in several autoimmune diseases, and its levels were elevated in macrophages [6–10]. In addition, Tougaard et al. [11] found that TL1A whole body knockout mice had a weight loss, decreased abdominal adipose tissue, and significantly decreased leptin expression in ileum and liver tissue. However, the role of TL1A-mediated regulation of macrophage polarization and recruitment in NASH is still unknown.

The authors found that the expression of TL1A was significantly increased in the livers of NASH murine models induced by high fructose high fat diets (HFHF) and methionine-choline deficiency (MCD) diets. Myeloid-specific TL1A overexpressed dramatically accelerated hepatic steatosis and M1-like phenotype macrophages infiltration in the liver. Moreover, bone marrow-derived macrophages (BMMs) and Kupffer cells (KCs) specific TL1A overexpressed promoted lipid accumulation and inflammatory factor secretion of nonalcoholic fatty hepatocytes *in vitro*.

## 2. Materials and Methods

### 2.1. Animals

The animal experiments were approved by Chinese Academy of Sciences Animal Care and Use Committee. Transgenic (Tg) mice with TL1A specific overexpression in myeloid cells were generated and genotyped as described [12] and provided by Cedars-Sinai Medical Center (America). They were bred and maintained in the specific pathogen-free condition at Experimental Animal Center of the Second Hospital of Hebei Medical University (China) and genotyped by real-time qPCR with tail DNA. At the same time, the littermate wild type (WT) mice were used as controls. Adult male mice of 6 ~ 8 weeks (weight: 18~21 g) were housed at 25°C ± 5°C and 12 h light/dark cycle with free access to water and food. The mice were fed with HFHF diets (76.5% normal chow, 12% lard, 1% cholesterol, 5% yolk powder, 5% whole milk powder, and 0.5% sodium cholate; Keao Xieli, China; 20% fructose, F8100, Solarbio, China) for 22 weeks, and a normal chow diet (Keao Xieli, China) for control. Besides, the MCD diet (A02082002B, Research Diets, USA) for 4 weeks was also used to construct the NASH model, and mice fed with methionine-choline-sufficient diet (A02082003B, Research Diets, USA) were regarded as controls.

### 2.2. Cell Culture

Bone marrow cells were isolated from the femur and tibia of WT and Tg mice, which were then incubated with macrophage colony-stimulating factor (M-CSF) for 10 days to differentiate BMMs. Afterwards, lipopolysaccharide (LPS) and interferon-*γ* (IFN-*γ*) were added into the culture medium to activate and differentiate BMMs into M1-like phenotype macrophages. Two days later, the medium as a conditional medium (CM) for hepatocytes culturing was collected.

KCs were harvested by liberase perfusion and differential centrifugation in Percoll [13]. Procedures of cell differentiation are as follows. Cells were isolated by perfusion of the liver using collagenase type IV. The parenchymal cells were removed by centrifuging (30 g/min × 5 min). Nonparenchymal sufficient supernatant was centrifuged in a 60%/30% Percoll gradient. The KC-enriched fraction was adjusted to 1 × 10^6^ cells/mL and plated in plastic culture plates for 2 h in the incubator (37°C, 5% CO2). Afterwards, nonadherent cells were removed by replacing the culture medium with fresh complete medium. Similarly, LPS and IFN-*γ* were added to make KCs activate and differentiate into M1-like phenotype macrophages, and then, the CM was collected after two days.

Primary hepatocytes were isolated as previously described [14]. Briefly, primary hepatocytes were isolated from the liver of male C57BL/6 mice by the collagenase perfusion method and then cultured in Dulbecco's modified Eagle's medium supplemented with 100 nmol/L dexamethasone and 1 g/mL insulin. The purity of hepatocytes detected by immunofluorescence staining was more than 90%. PA powder (P0500, Sigma-Aldrich, USA) and oleic acid (OA) (O1008, Sigma-Aldrich, USA) were dissolved in 0.1 M NaOH to make a stock solution, respectively. They were mixed at 2 : 1 as free fatty acid (FFA). To establish a cellular model of hepatic steatosis, the primary hepatocytes were exposed to an FFA mixture at a final concentration of 0.5 mM for 24 h. Finally, 50% CM from BMMs and KCs was used to incubate the nonalcoholic fatty hepatocytes.

### 2.3. Histological Analysis and Immunofluorescence Staining

Hematoxylin and eosin (H&E) staining and Oil red O staining were performed on paraffin-embedded and frozen liver sections, respectively. The expression of F4/80 (sc-52664, Santa Cruz, USA), TL1A (bs-5092R, Biosynthesis, China), Inducible Nitric Oxide Synthase (iNOS) (18985-1-AP, Proteintech, China), and CD206 (DF4149, Affinity, USA) was observed under confocal microscopy. The terminal deoxynucleotidyl transferase-dUTP nick end labeling (TUNEL) assay (C1086, Beyotime, China) was used to detect hepatocyte apoptosis.

### 2.4. Western Blot Analysis

TL1A (bs-5092R, Biosynthesis, China), CCR2 (DF7507, Affinity, USA), CXCR2 (DF7095, Affinity, USA), cleaved-caspase3 (AF7022, Affinity, USA), and cleaved-PARP1 (AF7023, Affinity, USA) were detected. The extraction of total protein was from liver tissues and macrophages. Equal amounts of total proteins were fractionated by SDS-PAGE, transferred onto polyvinylidene difluoride membranes, and then incubated with corresponding primary antibodies overnight at 4°C. Then, the membranes incubated with the secondary antibody, respectively, at 37°C for 1 hour.

### 2.5. Hepatic Lipid and Liver Function Assay

Liver function was evaluated in the serum and supernatant of hepatocytes by determining alanine aminotransferase (ALT) and aspartate aminotransferase (AST) using automatic biochemical analyzer (BECKMAN COULTER CX9, USA). Triglyceride (TG) and total cholesterol (TC) in liver tissue and primary hepatocytes were evaluated using the same analyzer.

### 2.6. Quantitative Real-Time PCR

Total mRNA from tissues and cells was extracted and then converted to cDNA (PC1802, Aidlab Biotechnologies, China). SYBR Green (FP205, TIANGEN, China) was used to perform quantitative real-time PCR amplification. The primers used in this study are presented in Supplemental Table 1.

### 2.7. Enzyme-Linked Immunosorbent Assay (ELISA) and Flow Cytometry

The TNF-*α*, IL-1*β*, IL-6, CCL2, and CXCL1 in serum and macrophage medium were measured using ELISA kits according to instructions of manufacturer (Multi Sciences, China). The antibody of F4/80 (11-4801-82), TNF-*α* (17-7321-82), pro-IL-1*β* (12-7114-82), and IL-6 (12-7061-82) and their isotype antibodies were all purchased from eBioscience and detected by FACS Verse flow-cytometer (BD Biosciences, USA). The specific steps were performed as previously reported [15]. Annexin V/PI was also detected by flow cytometry using Dead Cell Apoptosis Kit (V13242, Invitrogen, USA) according to instructions of manufacturer.

### 2.8. Statistical Analysis

All statistical analysis was accomplished using the SPSS 22.0 software. Data was expressed as means ± standard deviation (means ± SD). Differences between the two groups were determined using a two-tailed Student's *t*-test. Among multiple groups, differences were evaluated by one-way analysis of variance (ANOVA) followed by a Student-Newman-Keuls (SNK) post hoc test. *P* values less than 0.05 were considered to be statistically significant in all.

## 3. Results

### 3.1. TL1A Expression Is Upregulated in Liver Tissues and Macrophages of Mice with NASH

**Figure 1:**
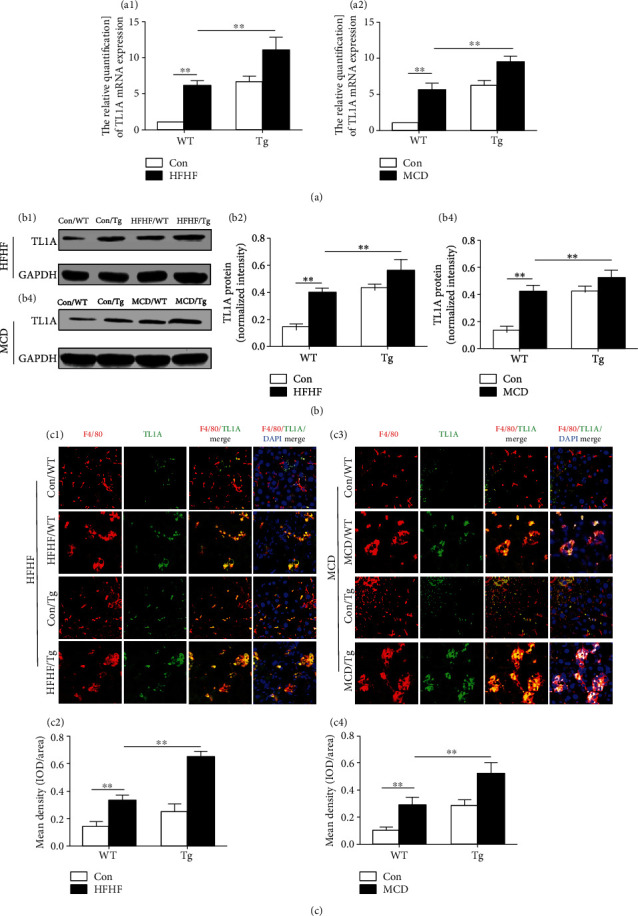
TL1A expression is upregulated in liver tissues and macrophages of mice with NASH. (a1) Relative mRNA levels of TL1A in mice of HFHF model were determined by quantitative real-time PCR (*n* = 6 per group). (a2) Relative mRNA levels of TL1A in mice of MCD model were determined by quantitative real-time PCR (*n* = 6 per group). (b1, b2) TL1A protein in mice of HFHF model was evaluated by Western blotting. (b3, b4) TL1A protein in mice of MCD model was evaluated by Western blotting. (c1, c2) TL1A protein of liver macrophages was detected by immunofluorescence double staining in mice of HFHF model (400x). (c3, c4) TL1A protein of liver macrophages was detected by immunofluorescence double staining in mice of MCD model (400x). Data are expressed as mean ± SD, ^∗∗^*P* < 0.01.

This paper evaluated the expression of TL1A in liver tissues and macrophages to explore the association between TL1A and NASH. For inducing steatohepatitis, adult male mice were fed HFHF diet for 22 weeks or MCD diet for 4 weeks. As shown in Figure 1(a1), the mRNA expression of TL1A in liver was upregulated in WT mice fed with HFHF diet compared with that in control mice (6.66 ± 0.68 vs. 1.00 ± 0.00, *P* < 0.01). HFHF-fed TL1A Tg mice had significantly higher TL1A expression than WT mice fed with the same diet (11.07 ± 1.56 vs. 6.66 ± 0.68, *P* < 0.01, Figure 1(a1)). As it was expected, mice fed with MCD also displayed more TL1A mRNA compared to control mice (*P* < 0.01), especially in MCD-fed Tg mice (Figure 1(a2)). Consistently, the protein expression of TL1A in liver samples from NASH mice was higher than that in control mice (Figure 1(b)). In addition, the expression of TL1A was significantly increased in liver macrophages of Tg mice than WT mice in control diet groups by immunofluorescence of liver sections (0.025 ± 0.006 vs. 0.015 ± 0.003, *P* < 0.01), and the difference was more significant in HFHF-fed mice (0.065 ± 0.003 vs. 0.033 ± 0.003, *P* < 0.01, Figures 1(c1) and 1(c2)). Consistent findings of elevated TL1A expression were confirmed using the MCD model (Figures 1(c3) and 1(c4)). Therefore, the authors reckoned that TL1A might participate in the pathology of NASH *via* hepatic macrophages.

### 3.2. Myeloid-Specific TL1A Overexpression Exacerbates Steatohepatitis

**Figure 2:**
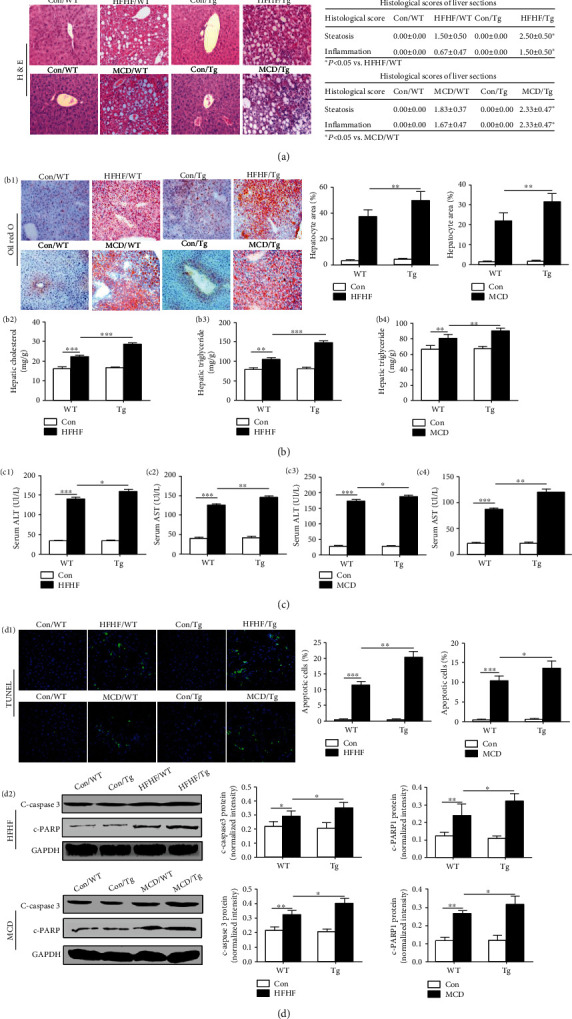
Myeloid-specific TL1A overexpression exacerbates steatohepatitis. (a) Representative liver H&E staining and histological scores of steatosis and inflammation of liver sections of mice with fed HFHF and MCD diets were showed (200x). (b1) Lipid droplets in liver sections of mice with fed HFHF and MCD diets were detected by Oil Red staining (200x). Hepatic cholesterol content (b2) and triglyceride content (b3) in mice of HFHF model and triglyceride content (b4) in mice of MCD model were test. (c1, c2) Serum ALT and AST in mice of HFHF model were measured. (c3, c4) Serum ALT and AST in mice of MCD model were measured. (d1) The percentage of apoptotic hepatocytes in mice of HFHF or MCD model was measured by TUNEL (400x). (d2) The expression of cleaved-caspase3 and cleaved-PARP in mice of HFHF and MCD model was detected by Western blotting to verify the level of apoptotic hepatocytes. Data are expressed as mean ± SD, ^∗^*P* < 0.05, ^∗∗^*P* < 0.01, ^∗∗∗^*P* < 0.001.

Myeloid-specific TL1A overexpressed Tg mice were used to investigate the effect of TL1A on liver injury and hepatocyte apoptosis. Compared to WT mice fed with HFHF or MCD, the significant steatohepatitis characterized by steatosis and inflammation was found in histological examination of liver sections of Tg mice fed with the same diet (Figure 2(a)). The increased hepatic steatosis in Tg mice with NASH compared to WT mice with NASH was confirmed by Oil Red O staining (Figure 2(b1)). Consistent with the pronounced steatosis seen from histology, hepatic cholesterol and hepatic triglyceride content was significantly higher in Tg mice with NASH compared to WT mice with NASH (Figures 2(b2)–2(b4)). In addition, serum alanine aminotransferase (ALT) and aspartic aminotransferase (AST) levels were markedly elevated in Tg mice fed with HFHF and MCD diet than WT counterparts (Figure 2(c)). Also, hepatocyte apoptosis was evaluated by TUNEL. It was found that hepatocyte apoptosis was significantly increased in HFHF-fed Tg mice compared to HFHF-fed WT mice (Figure 2(d1)). Consistent with the HFHF model, MCD-fed mice presented a similar result (Figure 2(d1)). Furthermore, these Western blot results were confirmed through detecting the protein of cleaved-caspase3 and cleaved-PARP (Figure 2(d2)). In summary, these results indicate that TL1A overexpression in myeloid cells exacerbates liver injury hepatocyte apoptosis in mice with NASH.

**Figure 3:**
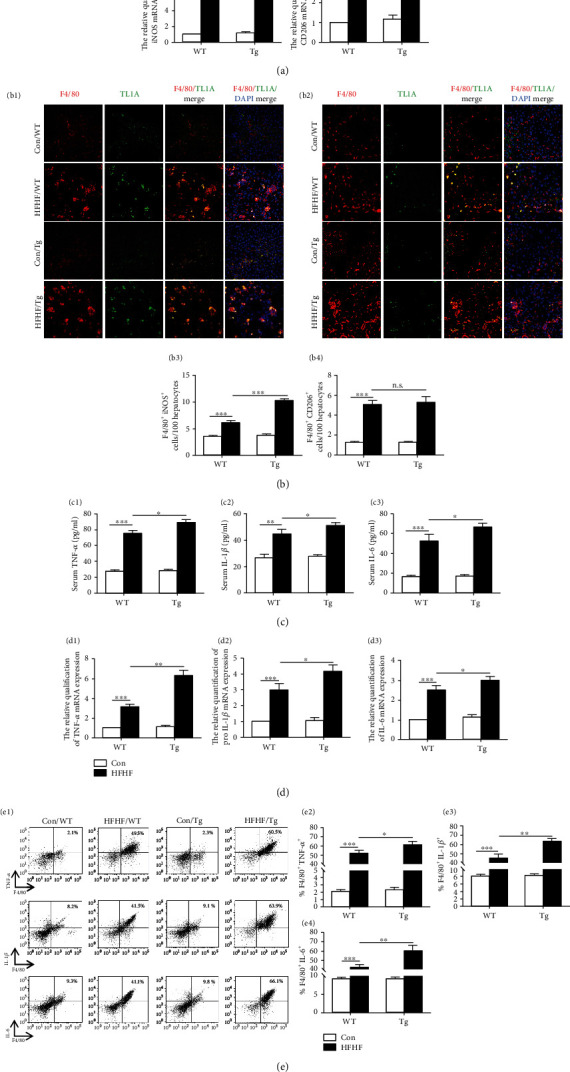
Myeloid-specific TL1A overexpression induces macrophages polarization to M1 phenotype. (a1) Relative mRNA levels of iNOS in mice of HFHF model were determined by quantitative real-time PCR. (a2) Relative mRNA levels of CD206 in mice of HFHF model were determined by quantitative real-time PCR. (b1, b3) iNOS protein of liver macrophages was detected by immunofluorescence double staining in mice of HFHF model (400x). (b2, b4) CD206 protein of liver macrophages was detected by immunofluorescence double staining in mice of HFHF model (400x). The expression of TNF-*α*, IL-1*β*, and IL-6 in serum (c1–c3), liver tissues (d1–d3), and liver macrophages (e1–e4) of mice fed with HFHF diets was detected by ELISA, quantitative real-time PCR, and flow cytometry, respectively. Data are expressed as mean ± SD, ^n.s.^*P* > 0.05, ^∗^*P* < 0.05, ^∗∗^*P* < 0.01, ^∗∗∗^*P* < 0.001.

iNOS and CD206 are markers of M1 and M2 macrophages, respectively. To characterize macrophage subtypes responsible for regulating NASH pathogenesis by myeloid-specific TL1A overexpression, iNOS and CD206 mRNA levels were analyzed. As shown in Figure 3(a1), the expression of iNOS mRNA in HFHF-fed Tg mice was significantly higher than HFHF-fed WT mice (8.11 ± 0.93 vs. 6.67 ± 0.95, *P* < 0.05, Figure 3(a1)). In contrast, the expression of CD206 mRNA was similar between HFHF-fed Tg mice and WT mice (2.59 ± 0.46 vs. 2.19 ± 0.39, *P* > 0.05, Figure 3(a2)). The expression of iNOS and CD206 mRNA in MCD model was consistent with the HFHF model (Supplementary Figures 1(a1) and 1(a2)). Furthermore, we detected F4/80/iNOS and F4/80/CD206 by Immunofluorescence double staining, and the mean integral optical density (IOD) of coexpression areas was analyzed. Compared to WT mice fed with the HFHF diet, Tg mice fed with the same diet showed that the IOD of F4/80+/iNOS+ was higher than WT mice (10.23% ± 0.37% vs. 6.04% ± 0.40%, *P* < 0.05, Figures 3(b1) and 3(b3)). However, the IOD of F4/80+/CD206+ was not changed (5.21% ± 0.56% vs. 5.03% ± 0.38%, *P* > 0.05, Figures 3(b2) and 3(b4)). Similarly, the IOD of F4/80+/iNOS+ and F4/80+/CD206+ in the MCD model was consistent with the HFHF model (Supplementary Figure 1(b)). These results confirmed M1 macrophage polarization in TL1A Tg mice of NASH.

Given that TNF-*α*, IL-1*β*, and IL-6 are the main proinflammatory factors secreted by M1 macrophages, those were also detected by ELISA in serum, quantitative real-time PCR in liver tissues and flow cytometry in hepatic macrophages to verify myeloid-specific TL1A overexpression could polarize macrophages towards to M1 phenotype. The levels of TNF-*α*, IL-1*β*, and IL-6 were upregulation in serum, liver tissues, and hepatic macrophages of Tg mice fed with HFHF diet compared to WT mice fed with the same diet (Figures 3(c)–3(e)). In agreement with this result shown, higher levels of TNF-*α*, IL-1*β*, and IL-6 in the serum were found in Tg mice fed with MCD for 4 weeks and liver tissue as well as liver macrophages compared to WT mice fed with the same diet (Supplementary Figures 1(c)-1(e)). These results collectively suggested that myeloid-specific TL1A overexpression exacerbates liver injury and hepatocyte apoptosis partly through promoting M1 macrophage polarization.

### 3.3. Myeloid-Specific TL1A Overexpression Promotes the Recruitment of Macrophages into the Liver in Mice with NASH

**Figure 4:**
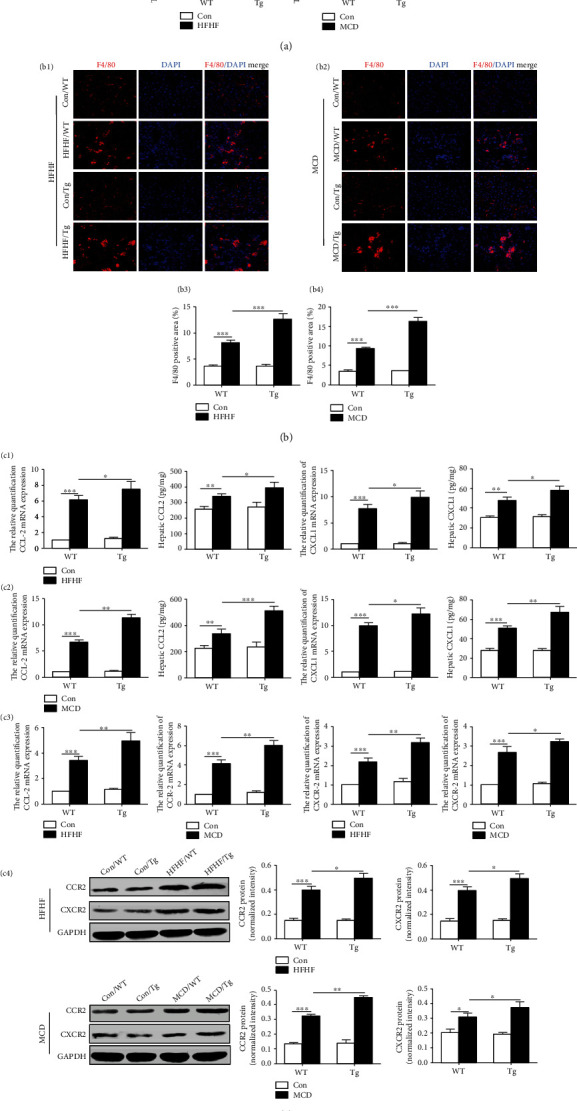
Myeloid-specific TL1A overexpression promotes the recruitment of macrophages into the liver in mice with NASH. (a1) Relative mRNA levels of F4/80 in mice of HFHF model were determined by quantitative real-time PCR. (a2) Relative mRNA levels of F4/80 in mice of MCD model were determined by quantitative real-time PCR. (b1, b3) F4/80 protein in mice of HFHF model was detected by immunofluorescence staining (400x). (b2, b4) F4/80 protein in mice of MCD model was detected by immunofluorescence staining (400x). (c1) The expression of CCL2 and CXCL1 in liver tissues of mice with fed HFHF diets was detected by quantitative real-time PCR and ELISA, respectively. (c2) The expression of CCL2 and CXCL1 in liver tissues of mice with fed MCD diets was detected by quantitative real-time PCR and ELISA, respectively. (c3) The expression of CCR2 and CXCR2 in liver tissues of mice with fed HFHF and MCD diets was detected by quantitative real-time PCR. (c4) The expression of CCR2 and CXCR2 in liver tissues of mice with fed HFHF and MCD diets was detected by Western blotting. Data are expressed as mean ± SD, ^∗^*P* < 0.05, ^∗∗^*P* < 0.01, ^∗∗∗^*P* < 0.001.

We subsequently investigated the effect of myeloid-specific TL1A overexpression on macrophages recruitment. As shown in Figures 4(a) and 4(b), the expression of F4/80 detected by quantitative real-time PCR and immunofluorescent staining in liver of Tg mice fed with HFHF and MCD was significantly increased compared to WT mice fed with the same diet (Figures 4(a) and 4(b)). It is known that as the potent chemoattractant, CCL2 and CXCL1 recruit a large number of CCR2-positive and CXCR2-positive monocytes from the blood to enter the liver in steatohepatitis, respectively [16, 17]. Therefore, hepatic macrophage infiltration was evaluated by detecting CCL2/CCR2 and CXCL1/CXCR2. Increased CCL2/CCR2 and CXCL1/CXCR2 in liver tissues of HFHF-fed Tg mice were confirmed. Consistent with the HFHF model, increased chemokines and their receptors in Tg mice fed with MCD displayed severe infiltration (Figure 4(c)). These results suggested that myeloid-specific TL1A overexpression could enhance macrophage infiltration contributing to steatohepatitis in mice.

### 3.4. Macrophages with TL1A Overexpression Induce Lipid Accumulation, Proinflammatory Cytokine Production, and Apoptosis in Hepatocytes In Vitro

**Figure 5:**
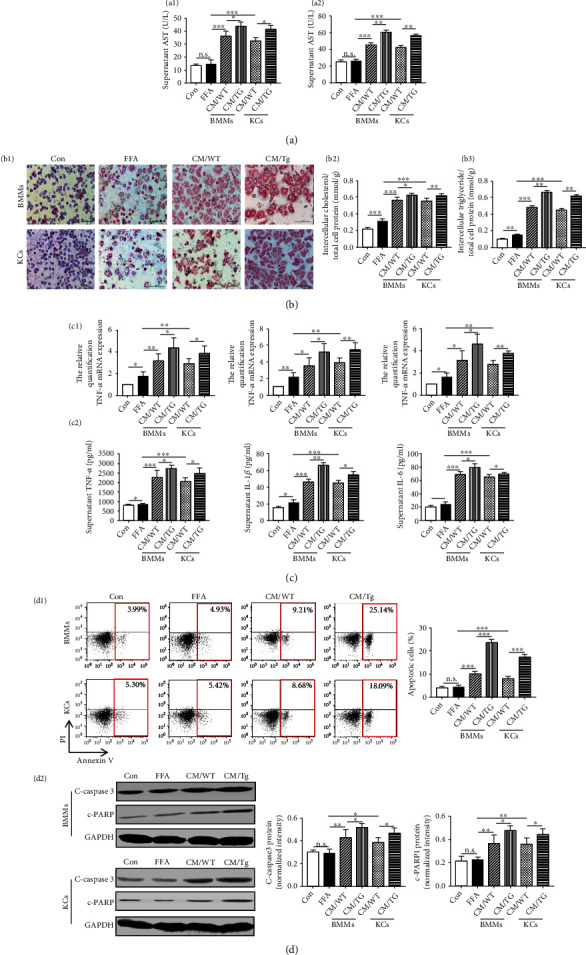
Macrophages with TL1A overexpression induce lipid accumulation, proinflammatory cytokine production, and apoptosis in hepatocytes *in vitro*. (a1, a2) Supernatant ALT and AST in hepatocytes were measured by automatic biochemical analyzer. (b1–b3) Lipid accumulation, intercellular cholesterol, and triglyceride content in primary hepatocytes were analyzed. The expression of TNF-*α*, IL-1*β*, and IL-6 in hepatocytes (c1) and these supernatant (c2) was detected by quantitative real-time PCR and ELISA, respectively. (d1) The percentage of apoptotic hepatocytes was measured by TUNEL. (d2) The expression of cleaved-caspase3 and cleaved-PARP in hepatocytes was detected by Western blotting. Data are expressed as mean ± SD, ^∗^*P* < 0.05, ^∗∗^*P* < 0.01, ^∗∗∗^*P* < 0.001.

Here, we explored whether TL1A overexpressed macrophages with M1 polarization can accelerate the steatohepatitis changes of hepatocytes *in vitro*. The supernatant of BMMs and KCs isolated from Tg and WT mice was collected as a conditional medium. Combined with FFA, it was used to incubate primary hepatocytes isolated from WT mice. The quantification of supernatant ALT and AST levels in hepatocytes was significantly increased in the cultured medium of macrophages from Tg mice compared to the medium of macrophages from WT mice (Figures 5(a1) and 5(a2)). Consistently, lipid accumulation (Figure 5(b)) and proinflammatory cytokines (TNF-*α*, IL-1*β*, and IL-6) (Figure 5(c)) of hepatocytes were also markedly increased in the cultured medium of TL1A overexpressed macrophages as compared to the medium of WT macrophages. In addition, TL1A-overexpressed macrophages conditioned medium promoted hepatocyte apoptosis as detected by Annexin V/PI and c-caspase3/c-PARP (Figure 5(d)). Collectively, the above data revealed that macrophages with TL1A overexpression could reinforce lipid accumulation, proinflammatory cytokine secretion, and apoptosis in hepatocytes *in vitro*.

### 3.5. TL1A Involves in M1 Macrophage Polarization and Recruitment via Proinflammatory Cytokines

**Figure 6:**
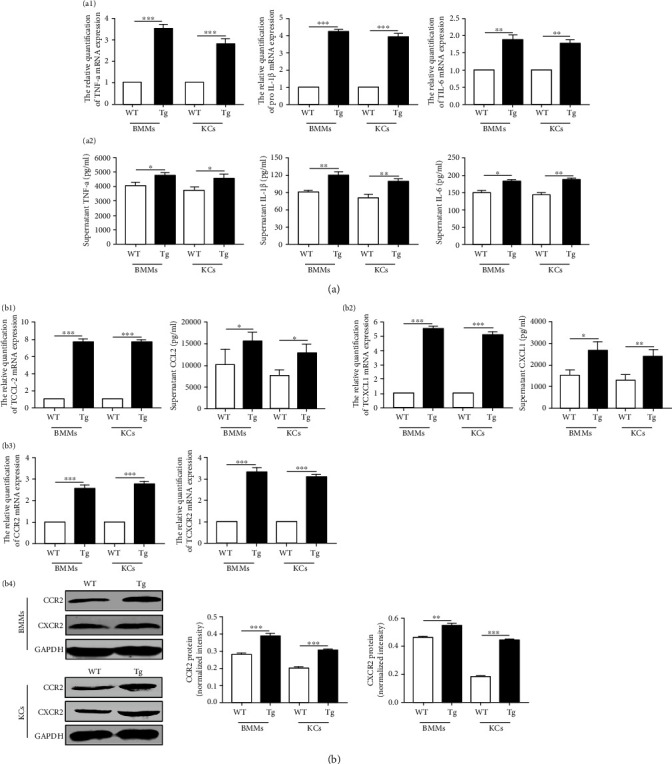
TL1A involves in M1 macrophage polarization and recruitment through the induction of cytokines. The expression of TNF-*α*, IL-1*β*, and IL-6 in BMMs and KCs (a1) and these supernatant (a2) was detected by quantitative real-time PCR and ELISA, respectively. (b1) The expression of CCL2 in BMMs and KCs and these supernatant was detected by quantitative real-time PCR and ELISA, respectively. (b2) The expression of CXCL1 in BMMs and KCs and these supernatant was detected by quantitative real-time PCR and ELISA, respectively. (b3) The expression of CCR2 and CXCR2 in BMMs and KCs was detected by quantitative real-time PCR. (b4) The expression of CCR2 and CXCR2 in BMMs and KCs was detected by Western blotting. Data are expressed as mean ± SD, ^∗^*P* < 0.05, ^∗∗^*P* < 0.01, ^∗∗∗^*P* < 0.001.

To assess whether TL1A could induce the polarization and recruitment of M1 macrophage, the cytokine profiles of the BMMs and KCs isolated from WT and Tg mice were analyzed. The expression of proinflammatory cytokines in macrophages was tested by quantitative real-time PCR and ELISA. As shown in Figure 6(a), TNF-*α*, IL-1*β*, and IL-6 were significantly increased in the BMMs from Tg mice compared to WT mice. Consistent with BMMs, the proinflammatory cytokines of KCs from Tg mice showed significantly higher than from WT mice. At the same time, the increased CCL2/CCR2 and CXCL1/CXCR2 in macrophages of Tg mice was also confirmed (Figure 6(b)). These results indicated that TL1A was involved in M1 macrophage polarization and recruitment through proinflammatory cytokines.

## 4. Discussion

The trend of increasing numbers of NAFLD worldwide has been alarming in recent years [18]. While NAFLD is mostly benign, NASH could lead to liver fibrosis and, ultimately, cirrhosis, increasing risks of hepatocellular carcinoma (HCC). It has been widely accepted that the M1/M2 balance of macrophages plays an important role in NASH. Intriguingly, the findings of this paper revealed a previously unknown function of TL1A in NASH pathogenesis by regulating M1 macrophages polarization, and it may be a new therapeutic target for the treatment of NAFLD. In this study, we found the enhanced expression of TL1A in liver tissues and macrophages using mouse models of NASH (Figure 1). Myeloid-specific TL1A overexpression increased the histological severity of steatohepatitis, lipid accumulation, and hepatocyte apoptosis. Our further observations showed that TL1A could promote the transformation of macrophages towards the M1 phenotype and the recruitment of macrophages into the liver in mice with NASH. These observations present the first evidence that TL1A might serve as a novel regulator in the pathogenesis of NASH.

TL1A involved in several immunological diseases, for example, rheumatoid arthritis, atherosclerosis, inflammatory bowel disease, and primary biliary cholangitis. Myeloid cells are one of the main mediators of TL1A. For instance, the authors also found that myeloid cells with TL1A overexpression induced the activation of macrophages and increased the secretion of inflammatory factors in liver fibrosis [10]. Furthermore, the TL1A knockout mouse has a striking metabolic phenotype characterized by weight loss, decreased abdominal adipose tissue, and tissue expression of leptin [11]. Currently, lots of studies have proved that TL1A plays a crucial role in regulating immunological disease, which includes the potential role of TL1A in the liver, especially in NAFLD. It needs further investigation at least under certain conditions. We found enhanced mRNA and protein expression of TL1A in liver tissues and macrophages under HFHF and MCD diet conditions. Furthermore, this study using myeloid-specific TL1A overexpression mice fed HFHF and MCD diets showed a decreased lipolysis and increased histological severity of steatohepatitis and hepatocyte apoptosis (Figure 2). These suggest that macrophage TL1A can contribute to hepatic steatosis, inflammation, and hepatocyte apoptosis.

Liver macrophage was identified as promoting the inflammation and fibrosis, as well as in the change from chronic damage to hepatoma [19]. Portal macrophage infiltration was found in human subjects with steatosis, and the number of liver macrophages was associated with the severity of liver steatosis and inflammation [20]. At the same time, KCs could initiate the liver inflammatory response by secreting inflammatory factors such as TNF-*α* and promote the recruitment of mononuclear macrophages to the liver to aggravate the liver inflammation in the mouse model of NASH [21]. Furthermore, KC depletion could protect against the insulin resistance, steatosis, and production of proinflammatory cell infiltration in NASH rats [22].

Under pathological conditions, liver macrophages manifest heterogeneity like steatohepatitis. Macrophages have two distinct phenotypes: inflammatory (M1) macrophages marked by iNOS participate in antigen recognition secreting inflammatory cytokines and noninflammatory (M2) macrophages marked by CD206 which are involved in tissue repair and remodeling. M1 macrophages contribute to the production of inflammatory cytokines that associated with the pathogenesis of hepatic steatosis and inflammation in the liver [23, 24]. Given that overexpression of TL1A in macrophages led to accelerated steatohepatitis, we hypothesized that macrophage polarization was inclined to the M1 phenotype when TL1A was overexpressed in macrophage. To prove this hypothesis, the authors counted the density of M1 and M2 macrophages of liver tissues from WT and myeloid-specific TL1A overexpression mice and compared the expression of inflammatory cytokines in macrophages with TL1A overexpression and normal macrophages *in vitro*. Compared with WT mice fed with HFHF/MCD, increased hepatic M1 macrophage markers (iNOS, TNF-*α*, L-1*β*, and IL-6) and unchanged M2 macrophage marker (CD206) were found in HFHF/MCD-fed myeloid-specific TL1A overexpression mice, which further confirmed our hypothesis (Figure 3 and Figure S1). The results of proinflammatory cytokines *in vivo* experiment were consistent with *in vitro* assays (Figures 6(a1) and 6(a2)). To sum up, results mentioned above suggested that macrophage TL1A overexpression exacerbated steatohepatitis at least in part due to M1 polarization.

Moreover, it has been confirmed that most of the liver macrophages which play a leading role in NASH are from mononuclear macrophages derived from hematopoietic stem cells in the bone marrow that are recruited through the circulation [16]. Once liver or intestinal fibrosis was formed, myeloid-specific TL1A overexpression mice showed more macrophages in the liver and intestine than WT mice [10, 12]. Similarly, the number of macrophages in hepatic tissues from Tg mice was significantly higher than that in WT mice. This result gave the evidence that the overexpression of TL1A in myeloid cells with NASH could contribute to recruitment of macrophages to liver. As efficient chemokines, CCL2 and CXCL1 could recruit a large number of CCR2 and CXCR1 positive mononuclear macrophages in the blood to enter liver. A large number of evidence have indicated that CCL2/CCR2 and CXCL1/CXCR2 played a critical role in recruiting macrophages into the liver in the progression of NAFLD [25–28].

Furthermore, *TL1A knockout* alleviated the expression of CXCL1 in the liver and ileum tissues and reduced the recruitment of inflammatory cells into the liver and ileum [11]. In this study, we found upregulated CCL2/CCR2 and CXCL1/CXCR2 in HFHF-fed and MCD-fed Tg mice. The above findings from these animal experiments were further confirmed by *in vitro* assays. The chemokines and receptors were increased in primary macrophages isolated from myeloid-specific TL1A overexpression mice compared to WT mice (Figure 6(b)). Therefore, our data indicated that the promotion of TL1A promoted the recruitment of macrophages which might be via the upregulation of CCL2/CCR2 and CXCL1/CXCR2 expression.

## 5. Conclusion

In conclusion, we described the functional role of TL1A in macrophage polarization and the recruitment of macrophages into the liver, which promotes the progression of steatohepatitis. These findings shed new light on TL1A-targeted strategy of NAFLD therapy, although more extensive studies are required.

## Data Availability

The data used to support the findings of this study are available from the corresponding author upon request.

## Supplementary Materials

Supplementary Materials Supplemental Table 1: oligonucleotide sequences used for quantitative real-time PCR analysis. Supplemental Figure 1: myeloid-specific TL1A overexpression induces macrophages polarization to M1 phenotype in mice of MCD model.
