# Supplementary material for: TNF-Like Ligand 1 Aberrance Aggravates Nonalcoholic Steatohepatitis via M1 Macrophage Polarization
Source: Oxid Med Cell Longev. 2021 Dec 31;2021:3877617. doi: 10.1155/2021/3877617 (PMC8741351; doi:10.1155/2021/3877617)
Supplement: Supplementary Materials — Supplemental Table 1: oligonucleotide sequences used for quantitative real-time PCR analysis. Supplemental Figure 1: myeloid-specific TL1A overexpression induces macrophages polarization to M1 phenotype in mice of MCD model. [file 3877617.f1.zip › 3877617.f1.docx]

**Supplementary**

TABLE 1 Oligonucleotide sequences used for quantitative real-time PCR analysis.

| Transcript |  | Sequence (5’-3’ direction) | Product size |
| --- | --- | --- | --- |
| TL1A | F  R | CGGGGAGACGACCAAACAAG  AAGGAGAACGTGGCCCCAAGGTAG | 160 bp |
| iNOS | F  R | CCACAAGGCCACATCGGATTTCA  AGGGGTAGGCTTGTCTCTGGGTC | 103 bp |
| CD206 | F  R | TGCCACTGCCATGCCTACCACAC  TGCCGTGCGTCTTGCCAGCTTTT | 132 bp |
| TNF-α | F  R | CCGCGACGTGGAACTGGCAGAAG  CCGATCACCCCGAAGTTCAGTAG | 150 bp |
| pro-IL-1β | F  R | ACAGATGAAGTGCTCCTTCCA  GTCGGAGATTCGTAGCTGGAT | 73 bp |
| IL-6 | F  R | CCACTTCACAAGTCGGAGGCTTA  GCAAGTGCATCATCGTTGTTCATAC | 112 bp |
| F4/80 | F  R | CCTGCCACAACACTCTCGGAAGC  TGGGCATGAGCAGCTGTAGGATC | 191 bp |
| CCL2 | F  R | TGCTTCTGGGCCTGCTGTTCACA  GCAGCAGGTGAGTGGGGCGTTAA | 89 bp |
| CCR2 | F  R | AGGCCATGCAGGTGACAGAGACTC  CCCCAGTGGAAGGAGTGAATGTAGAG | 204 bp |
| CXCL1 | F  R | CCGAAGTCATAGCCACACTCAA  GCAGTCTGTCTTCTTTCTCCGT | 128 bp |
| CXCR2 | F  R | CGGGTCATCTTCGCTGTCGTCCT  CGCGGCGCTCACAGGTCTCC | 118 bp |
| GAPDH | F  R | TCGTCCCGTAGACAAAATGG  TTGAGGTCAATGAAGGGGTC | 132 bp |

FIGURE S1 Myeloid-specific TL1A overexpression induces macrophages polarization to M1 phenotype in mice of MCD model. (a1) Relative mRNA levels of iNOS in mice of MCD model were determined by quantitative real-time PCR. (a2) Relative mRNA levels of CD206 in mice of MCD model were determined by quantitative real-time PCR. (b1 and b3) iNOS protein of liver macrophages was detected by immunofluorescence double staining in mice of MCD model (400x). (b2 and b4) CD206 protein of liver macrophages was detected by immunofluorescence double staining in mice of MCD model (400x). The expression of TNF-α, IL-1β and IL-6 in serum (c1-c3), liver tissues (d1-d3) and liver macrophages (e1-e4) of mice with fed MCD diets was detected by ELISA, quantitative real-time PCR and flow cytometry, respectively. Data are expressed as mean±SD, ^n.s.^*P*>0.05, **P*<0.05, ***P*<0.01, ****P*<0.001.
